# Factors associated with postpartum hemorrhage maternal death in referral hospitals in Senegal and Mali: a cross-sectional epidemiological survey

**DOI:** 10.1186/s12884-015-0669-y

**Published:** 2015-09-30

**Authors:** Julie Tort, Patrick Rozenberg, Mamadou Traoré, Pierre Fournier, Alexandre Dumont

**Affiliations:** Research Institute for Development, Paris Descartes University, Sorbonne Paris Cité, MERIT - UMR 216, Paris, France; UPMC University, Paris, France; Paris Diderot University, Paris, France; Department of Obstetrics and Gynecology, Poissy Saint-Germain Hospital, Poissy, France; EA7285, Clinical risk and safety in women’s health and perinatal health, University of Versailles Saint-Quentin (UVSQ), St Quentin en Yvelines, France; URFOSAME, Referral health center of the Commune V, Bamako, Mali; Research Centre of CHUM (CRCHUM), University of Montreal, Montreal, Canada; UMR 216, Faculté de Pharmacie, Laboratoire de Parasitologie, 4 Avenue de l’Observatoire, 75270 Paris Cedex 6, France

**Keywords:** Postpartum hemorrhage, maternal mortality, Sub-Saharan Africa

## Abstract

**Background:**

Postpartum hemorrhage (PPH) is the leading cause of maternal mortality in Sub-Saharan-Africa (SSA). Although clinical guidelines treating PPH are available, their implementation remains a great challenge in resource poor settings. A better understanding of the factors associated with PPH maternal mortality is critical for preventing risk of hospital-based maternal death. The purpose of this study was thus to assess which factors contribute to maternal death occurring during PPH. The factors were as follows: women’s characteristics, aspects of pregnancy and delivery; components of PPH management; and organizational characteristics of the referral hospitals in Senegal and Mali.

**Methods:**

A cross-sectional survey nested in a cluster randomized trial (QUARITE trial) was carried out in 46 referral hospitals during the pre-intervention period from October 2007 to September 2008 in Senegal and Mali. Individual and hospital characteristics data were collected through standardized questionnaires. A multivariable logistic mixed model was used to identify the factors that were significantly associated with PPH maternal death.

**Results:**

Among the 3,278 women who experienced PPH, 178 (5.4 %) of them died before hospital discharge. The factors that were significantly associated with PPH maternal mortality were: age over 35 years (adjusted OR = 2.16 [1.26–3.72]), living in Mali (adjusted OR = 1.84 [1.13–3.00]), residing outside the region location of the hospital (adjusted OR = 2.43 [1.29–4.56]), pre-existing chronic disease before pregnancy (adjusted OR = 7.54 [2.54–22.44]), prepartum severe anemia (adjusted OR = 6.65 [3.77–11.74]), forceps or vacuum delivery (adjusted OR = 2.63 [1.19–5.81]), birth weight greater than 4000 grs (adjusted OR = 2.54 [1.26–5.10]), transfusion (adjusted OR = 2.17 [1.53–3.09]), transfer to another hospital (adjusted OR = 13.35 [6.20–28.76]). There was a smaller risk of PPH maternal death in hospitals with gynecologist-obstetrician (adjusted OR = 0.55 [0.35–0.89]) than those with only a general practitioner trained in emergency obstetric care (EmOC).

**Conclusions:**

Our findings may have direct implications for preventing PPH maternal death in resource poor settings. In particular, we suggest anemia should be diagnosed and treated before delivery and inter-hospital transfer of women should be improved, as well as the management of blood banks for a quicker access to transfusion. Finally, an extent training of general practitioners in EmOC would contribute to the decrease of PPH maternal mortality.

## Background

Maternal mortality remains a major health problem worldwide, particularly in sub-Saharan Africa (SSA) where more than 50 % of maternal deaths occurred and where the lifetime risk of maternal death is 10 times higher than that in high-income countries [1]. Obstetric hemorrhage is the leading cause of maternal death in this region [2]. Most hemorrhages occur during the postpartum period [3] and are generally related to uterine atony, retained placenta and/or genital lacerations [4]. Clinical guidelines to treat post-partum hemorrhage (PPH) are available [5], but their implementation remains a great issue in resource poor settings.

There are three delays occurring during the diagnosis and management periods of PPH that are generally associated with a greater incidence in maternal mortality: delay in deciding to seek care (delay 1), delay in reaching the health facility (delay 2), delay in receiving quality emergency obstetric care (delay 3) [6]. Delay 3 is known to contribute more to maternal mortality than do delays 1 and 2 in SSA [6]. Late diagnoses, lack of drugs and supplies, referral delays, inadequate care or severe mismanagement and/or limited blood supply for transfusion are the most common problems observed [7] during the delay 3 in SSA [8].

In addition to this, the general maternal health status exerts a strong influence on the severity of PPH. For example, women presenting anemia, which is highly prevalent in SSA [9], are at better risk for severe PPH than others.

A better understanding of the factors associated with maternal death during PPH would help health care providers to rapidly identify women at highest risk for dying and provide appropriate care. To our knowledge, no previous studies have specifically addressed this question. Our objective was thus to identify the factors associated with maternal death occurring during PPH in referral hospitals in Senegal and Mali. The factors we tested were as follows: women’s characteristics, aspects of pregnancy and delivery; PPH management; and organizational characteristics in maternity units,

## Methods

### Setting

This is a cross-sectional study nested in the one-year pre-intervention period of a multicentre cluster-randomized trial (QUARITE trial). The objective of this trial was to evaluate the effectiveness of a multifaceted educational intervention for reducing hospital-based maternal mortality in Senegal and Mali.

46 public referral hospitals (22 in Mali and 24 in Senegal) with more than 800 deliveries a year and a functional operating room were recruited from Sept 1, 2007, to Oct 30, 2011. The trial consisted of a 1-year pre-intervention or baseline period, a 2-year intervention period, and a 1-year post-intervention period.

The study protocol has already been published [10, 11]. QUARITE trial is registered with ClinicalTrials.gov, number ISRCTN46950658. Ethical approval for QUARITE and this sub-study was granted by the ethics committee of Sainte-Justine Hospital in Montreal, Canada, and by the national ethics committees in Senegal and in Mali. The participating hospitals were included on the basis of informed consent by the local authorities. Collection of clinical data from hospital registers and medical records is authorized by the hospital authorities and does not require patient consent [11].

The 46 participant hospitals were representative of the existing public health referral system in both countries. The public health referral system, which is almost the only provider of modern health-care services in both countries, is based on district hospitals, regional hospitals, and national or teaching hospitals. These hospitals offer comprehensive emergency obstetric care in theory [12]. However, transfusion could be sometimes unavailable due to lack of blood products.

PPH cases are managed in the units where the complications arose. In district hospitals, the patients who require more specialized health care services (i.e. intensive care unit) are usually transferred to regional or national hospitals.

The intervention was not implemented during the baseline period of the trial. Therefore, there were no specific guidelines implemented by the research team during this period.

The participant hospitals represented 94 % of all referral hospitals in Mali and Senegal and covered approximately 10 % of all deliveries in both countries. According to available statistics in Mali and Senegal, among the remaining 90 % of deliveries, approximately 50 % occurred at home and 50 % in community health care centers in Mali and approximately 68 % occurred at home and 32 % in community health care centers in Senegal [13, 14].

### Population

All women who delivered in the participant hospitals during the study period were enrolled in the QUARITE trial.

In the present study, we only included women with PPH who delivered in participating hospitals during the baseline period (year 1, from October 2007 to October 2008). PPH was clinically assessed by the caregivers according to the visual estimation of excessive blood loss and patient status. The data collector recorded PPH only if the clinicians noted it in the clinical file. Women with uterine rupture or ante-partum hemorrhage (abruptio placentae or placenta praevia) or who died before delivery were excluded.

### Data collection

Characteristics of women, pregnancy, labor and delivery and PPH management were extracted from hospital registers and medical records and were registered every day by local trained data collectors (nurses or midwives) in a standard form. The standard form included a list of diagnoses related to the most frequent pathologies during pregnancy and delivery in Senegal and Mali and defined according to the Tenth International Classification of Diseases.

Hospital-based maternal deaths were identified among all the female deaths that occurred in the facility using the various registers available: admissions, hospitalizations (maternity and other services), operating rooms, and morgue. The cause of death was assessed by a doctor.

The quality and the completeness of the data were regularly controlled by the trial coordinator [11]. Missing data accounted for less than 1 % of cases. Available resources for each hospital were registered at inclusion by the trial coordinator using the standard inventory developed by WHO for the global survey for monitoring maternal and perinatal health [15]. This measure considers organizational data at the facility level and no data were missing.

### Study variables

The outcome was the hospital-based maternal death, measured as the vital status of the mother at hospital discharge categorized as alive or dead. All women transferred in another hospital after delivery were tracked by the data collectors or the national coordinator. The outcome was assessed in each case.

Four categories of potential risk factors for PPH maternal mortality were considered: characteristics of the women, and aspects of pregnancy and delivery before PPH; components of PPH management; and organizational characteristics of the hospitals. Well known individual risk factors were selected from relevant literature [16–19]. Institutional or contextual factors were selected from information based on our field experience and inputs from health providers in participating hospitals.

The maternal preexisting characteristics were as follows: country of residence (Mali or Senegal); location of the woman’s residence relative to the hospital in three categories: residence in the city of the hospital, outside the city of the hospital but in the same region, outside the region of the hospital; maternal age at delivery in three categories: <20, 20–35 and > 35 years.

Aspects of pregnancy, labor and delivery include: parity and previous cesarean delivery categorized as: nulliparous, multiparous without cesarean delivery, multiparous with cesarean delivery (one or more); number of prenatal visits categorized as: 0, 1–4, >4; multiple pregnancy; preexisting diseases before pregnancy including: positive serology of human immunodeficiency virus (HIV), chronic pulmonary, cardiac or renal diseases, sickle cell trait or chronic hypertension; prepartum severe anemia (<7 g/dL); gestational hypertensive disorders including the following diagnoses: gestational hypertension, pre-eclamspia, eclampsia, hemolysis, elevated liver enzymes and low platelet count (HELLP) syndrome ; referral from another health facility; induction of labor; prolonged labor, including the following diagnoses: obstructed labor, cephalopelvic disproportion, dystocia, labor not progressing according to a normal partogram; mode of delivery categorized as: spontaneous vaginal delivery, C-section before the onset of labor, C-section after the onset of labor, forceps/vacuum extraction; and birth weight categorized as: <2500, 2500–4000, >4000 grs.

Three components of PPH management were analyzed: transfusion of blood products (fresh blood or red blood cells), hysterectomy and transfer after delivery to another hospital if the patient required more specialized health care services.

The organizational characteristics of the hospitals where the women gave birth included: the hospital type classified as hospital in the capital, regional hospital outside the capital or district hospital outside the capital; skilled staff for cardiopulmonary maternal resuscitation or hysterectomy; physician specialized in anesthesia; the qualification of the physician for obstetric care on staff categorized as: gynecologist-obstetrician, when a specialist is available in the hospital or general practitioner (GP) trained in emergency obstetric care (EmOC), when a trained GP is available but no gynecologist-obstetrician; availability of a blood bank, adult intensive care unit, peripartum and postpartum care guidelines or continuous medical training program.

### Statistical analysis

Descriptive statistics were conducted to portray women’s characteristics, aspects of pregnancy and delivery; PPH management; and organizational characteristics of hospitals. Then, the maternal mortality proportion was calculated overall and for each factor cited above.

To test the association between each factor and PPH maternal mortality, we used a series of random-intercept hierarchical logistic regression models to take into account the dependence of observations within hospitals.

A three-stage statistical procedure was used. First, a series of univariate hierarchical logistic regressions were performed to identify which factor was associated with PPH maternal mortality. The association was quantified with unadjusted odds ratios and their 95 % confidence intervals. A critical p value inferior to 20 % was chosen for variable selection in further analyses.

Second, a multivariate hierarchical logistic regression was performed using women’s characteristics, aspects of pregnancy and delivery that were selected in the first step. A critical p value inferior to 5 % was used for significance (model 1).

Third, a multivariate hierarchical logistic regression was performed using components of PPH management and controlling by the variables significant in model 1. A critical *p* value inferior to 5 % was used for significance (model 2).

Finally, a multivariate hierarchical logistic regression was performed using organizational characteristics and controlling by the variables significant in model 2 (model 3).

The relevant interactions between any two variables were tested with the Wald test.

Cases with one or more missing values among the characteristics of women, delivery and PPH management were not included in the multivariate analyses (*n* = 49 Women, 1,5 % of total).

We estimated the relative contribution of characteristics of women, PPH management factors and institutional factors to the variability of maternal mortality between hospitals. To that purpose, we used the ratios of the random intercept variances [20].

The data were analyzed with Stata v.12 Software (Stata Corporation, College Station, TX).

## Results

**Fig. 1:**
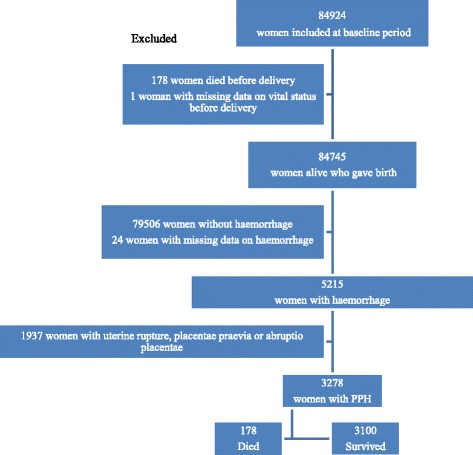
Flow chart

Among 84,924 women who delivered in participant hospitals and were included in the QUARITE trial during the pre-intervention period, 3,278 met the inclusion criteria (Fig. 1). Among these women with PPH, 178 (5.4 %) died in the postpartum period and before hospital discharge. These proportions varied considerably between hospitals from 0 to 23.1 % (data not shown).

### Selection of women’s characteristics, aspects of pregnancy and delivery

The distribution of women’s characteristics, aspects of pregnancy and delivery and their association with PPH maternal mortality are presented in Table 1. In multivariate analysis, age over 35 years, residence in Mali and outside the region where the hospital is located, pre-existing diseases before pregnancy, severe chronic anemia, birth weight over 4000 grs and forceps or vacuum delivery were positively associated with risk of maternal death under PPH.

**Table 1:** Characteristics of women, pregnancy and delivery: distribution in the cohort of PPH and risk of death. Univariate and multivariate analysis

	Number	Percent	CFR* (%)	OR	95 % CI	aOR^a^	95 % CI
Women and pregnancy							
Country of residence							
Senegal	2551	77.8	4.8	1.00	-	1.00	-
Mali	727	22.2	7.7	1.56	1.05–2.31	1.84	1.13–3.00
Location of residence							
In the city of the hospital	2058	63.5	5.0	1.00	-	1.00	-
Outside the city but in the same region	982	30.3	5.1	1.18	0.77–1.80	1.49	0.91–2.44
Outside the region	203	6.3	10.8	2.25	1.30–3.89	2.43	1.29–4.56
Age							
Less than20 years	649	19.8	4.2	1.00	-	1.00	-
Between 20 and 35 years	2094	63.9	5.3	1.34	0.87–2.07	1.48	0.93–2.44
More than35 years	535	16.3	7.5	1.88	1.13–3.13	2.16	1.26–3.72
Parity							
Nulliparous	691	21.1	5.5	1.00	-		
Multiparous without previous caesarean	2436	74.3	5.4	0.99	0.68–1.44		
Multiparous with previous caesarean	151	4.6	8.0	0.90	0.41–1.98		
Number of prenatal visit							
No visit	510	15.8	7.5	1.47	0.99–2.17	1.14	0.74–1.74
Between1 and 4	2514	78.0	4.8	1.00	-	1.00	-
More than4	201	6.2	6.5	1.38	0.76–2.52	1.39	0.74–2.61
Preexisting disease before pregnancy^b^	17	0.5	35.3	9.47	3.35–26.73	7.54	2.54–22.44
Multiple pregnancy	184	5.6	5.4	1.00	0.52–1.96		
Prepartum severe anemia (<7 g/dL)	85	2.6	25.9	7.05	4.15–11.98	6.65	3.77–11.74
Gestational hypertensive disorders^c^	406	12.4	7.4	1.69	1.10–2.59	1.57	0.98–2.52
Referral from another health facility	2045	62.4	5.9	1.32	0.95–1.84	1.10	0.77–1.57
Labor and delivery							
Induction of labor	554	16.9	4.3	0.84	0.53–1.35		
Prolonged labor	710	21.7	7.8	1.64	1.15–2.33	1.21	0.79-1.84
Mode of delivery							
Spontaneous vaginal	2751	84.2	4.7	1.00	-	1.00	-
C-section after the onset of labor	316	9.7	8.2	1.77	1.13–2.78	1.54	0.91–2.61
C-section before the onset of labor	124	3.8	7.3	1.69	0.83–3.46	1.86	0.89–3.91
Forceps/Vacuum	77	2.4	13.0	2.80	1.39–5.68	2.63	1.19–5.81
Birth weight							
Less than 2500 grs	1594	48.6	4.8	1.00	-	1.00	-
2500–4000 gs	1600	48.8	5.3	1.09	0.79–1.50	1.02	0.72–1.44
More than 4000 grs	84	2.6	20.2	4.88	2.69–8.84	2.54	1.26–5.10
Total	3278	100.0	5.4				

*CFR: Case fatality rate. ^a^aOR: Odds Ratio and 95 % confidence interval, adjusted for country of residence, location of residence, age, number of prenatal visit, preexisting disease before pregnancy, prepartum severe anemia, gestational hypertensive disorders, referred from another health facility, prolonged labor, mode of delivery and birth weight (*N* = 3179 PPH). Missing data: Location of residence (*n* = 35), number of prenatal visit (*n* = 53), induction of labor (*n* = 7), referred from another health facility (*n* = 1), mode of delivery (*n* = 10). ^b^Preexisting disease before pregnancy: HIV, chronic pulmonary, cardiac or renal diseases, sickle cell trait or chronic hypertension. ^c^Gestational hypertensive disorders: gestational hypertension, preeclampsia, eclampsia, HELPP syndrome

### Components of PPH management

Transfusion was administered in 27.7 % of women with PPH (Table 2). Some patients required a hysterectomy (1.1 %) or a transfer to another hospital (1.1 %). After adjustment for women’s characteristics, aspects of pregnancy and delivery, the risk of death for women with PPH was 2.17 higher in women with transfusion, and 13.35 higher in women who required a transfer to another hospital. One-half of the transferred women died during transportation or after the admission to the other hospital. Hysterectomy was associated with an increased risk of case fatality, but the association was not significant when controlling for other factors.

**Table 2:** Components of PPH management: distribution in the cohort of PPH and risk of death, univariate and multivariate analysis

	Number	Percent	CFR* (%)	OR^a^	95 % CI	aOR^a^	95 % CI
Transfusion	907	27.7	9.0	2.67	1.94–3.69	2.17	1.53–3.09
Hysterectomy	35	1.1	17.1	3.64	1.45–9.11	2.18	0.79–6.02
Transfer to another hospital	36	1.1	50.0	19.80	9.85–39.84	13.35	6.20–28.76

*CFR: Case fatality rate. ^a^aOR: Odds Ratio and 95 % confidence interval, adjusted for country of residence, location of residence, age, preexisting disease before pregnancy, prepartum severe anemia, mode of delivery, birth weight, transfusion, hysterectomy and transfer to another hospital (*N* = 3227 PPH). Missing data: transfusion (*n* = 3), hysterectomy (*n* = 4), transfer to another hospital (*n* = 3)

### Organizational characteristics of hospitals

The distribution of hospital characteristics is shown in Table 3, as well as their associations with PPH maternal death. More than half of women who experienced PPH were managed in regional hospitals, with a blood bank available, skilled staff for transfusion, cardiopulmonary maternal resuscitation and hysterectomy, and with guidelines and continuous medical training available. However, less than 50 % of women were treated in hospitals with a physician specialized in anesthesia on staff and with an adult intensive care unit available. More than 80 % of women were treated in a maternity unit with a gynecologist-obstetrician on staff.

**Table 3:** Characteristics of the hospitals: distribution in the cohort of PPH and risk of death, univariate analysis and multivariate analysis

	Number	Percent	CFR* (%)	OR	95 % IC	aOR^a^	95 % IC
Type of hospital							
Hospital in the Capital	655	20,0	4.3	1.00	-		
Regional hospital outside the capital	1668	50.9	5.3	1.30	0.76–2.22		
District hospital outside the capital	955	29.1	6.5	1.69	0.98–2.93		
Specialized care services availability							
Blood bank	1982	60.5	5.9	1.27	0.84–1.92		
Adult intensive care unit	1516	46.2	5.5	1.07	0.71–1.63		
Human resource availability							
Skilled staff for cardiopulmonary resuscitation	1886	57.5	4.8	0.73	0.50–1.09	0.98	0.65–1.47
Skilled staff for hysterectomy	3232	98.6	5.4	0.66	0.18–2.43		
Qualification of physician for obstetric care on staff							
Trained general practitioner	558	17.0	9.7	1.00	_	1.00	_
Gynecologist-obstetrician	2720	83.0	6.1	0.45	0.32–0.62	0.55	0.35–0.89
Physician specialised in anaesthesia	1323	40.4	4.5	0.69	0.45–1.05	0.87	0.57–1.34
Protocol and training							
Intrapartum care guidelines	2650	80.8	5.5	1.19	0.68–2.06		
Postpartum care guidelines	2650	80.8	5.5	1.19	0.68–2.06		
Continuous medical training	2562	78.2	5.5	1.12	0.67–1.87		

*CFR: Case fatality rate. ^a^aOR: Odds Ratio and 95 % confidence interval, adjusted for country of residence, location of residence, age, pre-existing diseases before pregnancy, prepartum severe anemia, mode of delivery and birth weight; transfusion and transfer to another hospital; and for gynecologist-obstetrician, physician specialist in anesthesia, skilled staff for cardio-pulmonary resuscitation (*N* = 3229 PPH). Type of hospital is not included because it is highly correlated with the qualification of physician for obstetric care on staff

In an univariate analysis, “gynecologist-obstetrician on staff” was significantly associated with a decrease risk of death (*p* < 0.05) (Table 3). After adjustment for all characteristics selected in the precedent models, this association remained significant. Hospitals having a gynecologist-obstetrician were associated with a decreased risk of death by 45 % (aOR = 0.55; 95 % IC: [0.35–0.89]; *p* = 0.014) when compared to hospitals that did not have.

Maternal characteristics, components of PPH management and organizational characteristics of hospitals that were included in the final model explained respectively 2.7, 1.4 and 10.9 % of the between-hospital variability of maternal mortality (85 % residual variability).

## Discussion

To our knowledge, the present paper is the first to document the factors associated with PPH maternal death in referral hospitals in SSA. Our findings suggest that some women’s characteristics, aspects of pregnancy and delivery; PPH management; and organizational characteristics of hospitals are determinants of hospital-based PPH maternal death. In particular, these include the level of accessibility to the hospital, woman’s health status before PPH, the mode of delivery and birth weight. Availability of a gynecologist-obstetrician on staff is associated with a decreased risk of death, while transfusion and inter-hospital transfer are linked to an increased risk of death.

Our study showed that living in Mali is associated with a higher risk of PPH maternal mortality than in Senegal. No difference in the characteristics of women, pregnancy and childbirth between the two countries could explain this result. However, the prevalence of PPH in our study is higher in Senegal than in Mali. PPH definition might differ between the two countries. In Senegal, PPH diagnosis is mainly based on visual estimation of blood loss (i.e. estimation over 500 mL), while in Mali, this is mainly based on visual estimation and patient status. The definition in Mali may lead to detect more severe PPH cases than in Senegal. This could explain why the case fatality rate is higher in Mali than in Senegal. In addition, the number of regional hospitals participating to the QUARITE Trial is higher in Senegal [11] than in Mali [4], while the number of district hospitals is higher in Mali [12] than in Senegal [7]. Health care professionals are globally more qualified in regional hospitals than in district hospitals, and they are able to diagnose PPH cases more likely in the higher level of care.

Our study showed that several women’s characteristics, aspects of pregnancy and delivery are associated with PPH maternal mortality. This could help care providers in referral hospitals in Mali and Senegal to strengthen both diagnosis and treatment of PPH among women at high risk. Some characteristics are already known as risk factors for severe PPH: age over 35 years old, pre-existing disease before the occurrence of PPH including severe anemia, instrumental delivery and birth weight over 4000 g [16–19]. Chronic diseases alter the physiological health status of the woman and increase the risk of organ dysfunctions when a hemorrhagic complication arises [21]. Moreover, a forceps or vacuum-based delivery or a birth weight greater than 4000 g increases the use of episiotomy or vaginal lacerations and thus more blood loss [22, 23]. From this arose the need of careful monitoring during immediate post-partum period.

The level of accessibility to referral hospitals is also a well-known risk factor for maternal death in SSA [6]. Long transport times between home and health care facility, increase the odd of poor health status of the women when admitted to the referral hospital. The inherent issues of communication existing between the health care facilities in SSA [6] also increase care delays and may lead to dramatic high case fatality rate (50 % in our study). In such a context, it is necessary to improve identification of women who require transfer, to ensure an efficient transfer organization and the quality of transportation. Particularly, the non-pneumatic anti-shock garment could make a significant contribution to help women survive delays due to transfer [24].

Paradoxically, our findings showed transfusion is associated with a higher risk of maternal death. Transfusion is however an essential care in the management of PPH. It helps to maintain appropriate blood pressure in case of significant blood loss. This association may thus reflect an indication bias because transfusion is more likely performed among women presenting the most severe PPH. From this, we believe that a significant proportion of maternal deaths was probably related to severe PPH and that transfusion may have been performed too late or with insufficient quantity of blood products [8].

In our study, a gynecologist-obstetrician on staff is significantly associated with a decreased of risk of PPH maternal mortality. When a physician specialist is not available in the hospital, a trained general practitioner (GP) is in charge for the maternity unit. These results suggest that training in emergency obstetric care of GPs is probably insufficient. In both countries this training essentially allows to overcome the lack of obstetrician in the district hospitals. However, this training is informal and lasts over a few months. There is no examination or certification of achievement or degree. Knowledge of these physicians is thus limited and may not allow them to quickly diagnose PPH and provide quality care. This can also increase delays in decision-making and particularly the use of surgical treatments (arterial ligation, hysterectomy). Our findings confirm that the GPs are more likely employed in district hospitals than obstetricians, where a small annual amount of deliveries occur (data not shown), with less qualified care providers and limited drugs and supplies. The QUARITE trial showed that health care professionals training in emergency obstetric care had led to a significant reduction in maternal mortality in the district hospitals [10].

This study presents several strengths. Data were collected from 46 referral hospitals in Senegal and Mali, which were representative of the existing health system in both countries. Thus, this approach ensures the external validity of the study, whose findings could be easily generalized to health systems with similar characteristics. Sample size was large enough to perform hierarchical analyses with high statistical power (*N* = 3278 women). Moreover, information on the vital status of women came from various sources, as a consequence, the number of hospital-based maternal deaths was considered exhaustive.

However, our study also presents some limitations. First, blood loss was measured only with a subjective method by care givers and PPH may have been underestimated in our study [25]. The reported rate of PPH in our study was relatively small with 3.9 %. That rate was lower than that observed in African health care facilities as shown in Calvert et al. [25]. Second, it remains possible that diagnoses were under-reported in medical files. That bias may reduce the strength of the association between studied maternal characteristics and PPH case fatality. Third, we did not assess if the care providers were aware of the active management of the third stage of labor recommendations for PPH prevention and whether or not it was implemented. Fourth, a large part of the between-hospital variability in PPH maternal mortality remained unexplained. This heterogeneity may be due to differences in medical practices that were not measured in our study. Younger physicians working alone in rural settings may have different practices than older ones working in urban settings. Taking this information into account would improve the performance of our predictive model. Finally, most women in this study were discharged from the hospital two days or less after delivery. Maternal deaths occurring at home were not recorded. Data analyzed included information only from women who sought care at participant facilities, and excluded information on those delivered at home or referred from another health care facility after delivery. Therefore, maternal mortality in the population cannot be inferred and these results should be used only by clinicians to reduce hospital-based mortality related to PPH.

## Conclusions

Our findings may have direct implications for clinicians in resource poor settings. We demonstrated that women’s characteristics, aspects of pregnancy and delivery; PPH management; and organizational characteristics of hospitals are risk factors for PPH maternal mortality in SSA. We suggest that severe antepartum anemia should be diagnosed more accurately and treated before delivery, while effort should be put on improving the capacity of the blood banks and making blood quickly accessible when needed. Early detection of PPH and timely decision-making for transfusion are critical. It is also important to improve the identification of women who need to be transferred to another facility and transportation system. Finally, our findings suggest that training in emergency obstetric care of GPs is probably insufficient. If that training may respond to the shortage of obstetricians, whose training is time and money consuming; on the other hand it prevents the same quality care. A better supervision and extensive training are essential keys to reduce gaps in knowledge and to prevent PPH maternal death.
